# Hijacking Chemical Reactions of P450 Enzymes for Altered Chemical Reactions and Asymmetric Synthesis

**DOI:** 10.3390/ijms24010214

**Published:** 2022-12-22

**Authors:** Eerappa Rajakumara, Dubey Saniya, Priyanka Bajaj, Rajanna Rajeshwari, Jyotsnendu Giri, Mehdi D. Davari

**Affiliations:** 1 Macromolecular Structural Biology Lab, Department of Biotechnology, Indian Institute of Technology Hyderabad, Kandi, Sangareddy 502284, India; 2 Department of Chemical Sciences, National Institute of Pharmaceutical Education and Research (NIPER), NH-44, Balanagar, Hyderabad 500037, India; 3 Department of Plant Pathology, College of Horticulture, University of Horticultural Sciences, Bagalkot Campus, GKVK, Bengaluru 560064, India; 4 Department of Biomedical Engineering, Indian Institute of Technology Hyderabad, Kandi, Sangareddy 502284, India; 5 Department of Bioorganic Chemistry, Leibniz Institute of Plant Biochemistry, Weinberg 3, 06120 Halle, Germany

**Keywords:** prosthetic group, stereoselectivity, regioselectivity, asymmetric synthesis, cytochrome P450, enzyme engineering, biocatalysis

## Abstract

Cytochrome P450s are heme-containing enzymes capable of the oxidative transformation of a wide range of organic substrates. A protein scaffold that coordinates the heme iron, and the catalytic pocket residues, together, determine the reaction selectivity and regio- and stereo-selectivity of the P450 enzymes. Different substrates also affect the properties of P450s by binding to its catalytic pocket. Modulating the redox potential of the heme by substituting iron-coordinating residues changes the chemical reaction, the type of cofactor requirement, and the stereoselectivity of P450s. Around hundreds of P450s are experimentally characterized, therefore, a mechanistic understanding of the factors affecting their catalysis is increasingly vital in the age of synthetic biology and biotechnology. Engineering P450s can enable them to catalyze a variety of chemical reactions viz. oxygenation, peroxygenation, cyclopropanation, epoxidation, nitration, etc., to synthesize high-value chiral organic molecules with exceptionally high stereo- and regioselectivity and catalytic efficiency. This review will focus on recent studies of the mechanistic understandings of the modulation of heme redox potential in the engineered P450 variants, and the effect of small decoy molecules, dual function small molecules, and substrate mimetics on the type of chemical reaction and the catalytic cycle of the P450 enzymes.

## 1. Introduction

C=C and C–H functionalization based transition metal-catalyzed oxene, carbene, and nitrene transfer reactions in stereo- and regio-specific manners in a broad spectrum of substrates, are widely used to synthesize natural product intermediates, pharmaceuticals, and fine chemicals [1,2]. A heme-prosthetic group containing enzymes such as P450 monooxygenases (P450 or CYP), heme peroxidases, and peroxygenases exhibit a broad range of naturally catalyzed reactions such as hydroxylation, nitration, carbon-carbon bond cleavage, oxidative dealkylation, heteroatom oxidation, epoxidation, and oxidative ring coupling through an initial oxene transfer in a regio- and stereoselective manner [1,2,3]. Some unusual reactions, such as chlorine oxygenation, aromatic dehalogenation, desaturation, oxidative aryl migration, etc., are also catalyzed by P450s [4]. Some oxene-transferring P450s have evolved to catalyze the carbene and nitrene transfer reactions through directed evolution. Though P450s catalyze a myriad of oxidative transformations, the industrial application is limited due to low activity, low organic solvent tolerance, the need for a costly cofactor and its regeneration, lack of stability, low coupling efficiency, lack of required combinations of substrate specificity, regio- and stereoselectivity, and chemoselectivity [1,5]. Understanding the structure–function relationship, the mechanistic basis for catalytic cycles of non-native functional group transfer, rearrangements of oxidative products, rearrangements involving enzyme intermediates, the effect of modulation of heme redox potential on the catalytic cycle, reaction rate, chemoselectivity, and cofactor requirement would help to tailor the P450s for industrial requirements. This understanding is also helpful for engineering P450s for chemoselectivity, catalytic efficiency, switching the cofactor requirement, biotransformation applications, regioselectivity, and asymmetric synthesis. Herein, we provide an intersectional overview of key aspects of P450 enzymatic reactions, thermodynamics of heme redox potential modulation through rational engineering linked to the rate of the reaction, the rate of the formation of intermediates, stability and life span, abrogation of a native chemical reaction while enabling non-native reactions, stereoselectivity, and the formation of reaction intermediates independent of substrate binding. We also discuss how creating substrate-binding pockets and engineering subtle variations in the heme coordination residues convert hemoglobin into stereo- and regioselective biocatalysts. This review also reports recent advances in the manipulation of P450 chemical reactions using the substrate-mimicking decoy and dual-function small molecules for adapting the P450-mediated peroxide shunt pathway for regio- and stereoselective sulfoxidation, hydroxylation, epoxidation, and oxidative demethylation catalytic reactions for the cost-effective use of biocatalysts [6,7]. Finally, we critically discuss the rational engineering of nicotinamide adenine dinucleotide phosphate (NADPH)-dependent monooxygenases into H_2_O_2_-peroxygenases, which uses low-cost H_2_O_2_ compared to high-cost NADPH as the cofactor [8,9].

## 2. Factors Affecting the Heme Redox Potential

**Figure 1:**
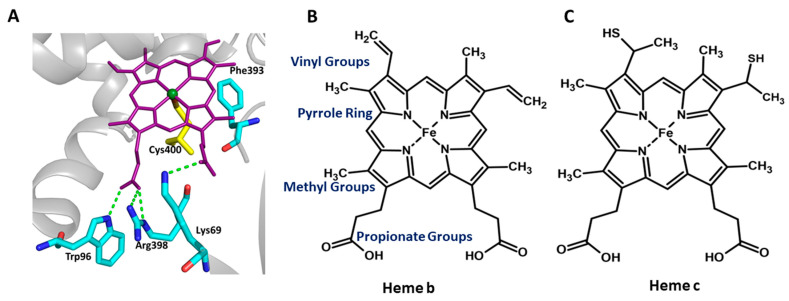
The catalytic pocket of the representative P450 (P450_BM3_) enzyme and the schematic representation of heme prosthetic groups present in the P450 and non-enzyme proteins. (**A**) Protoporphyrin IX, the prosthetic group of Cytochrome P450 (purple sticks), interacts with Trp96, Arg398, and Lys69 (Cyan sticks). Cys400 (yellow sticks) is a highly conserved residue that forms the heme–thiolate bond. Phe393 is present within the heme-binding motif of P450_BM3_, and positioned close to the Cys400, and is represented by cyan sticks. (**B**,**C**) Comparison of different heme groups. Heme b is the prosthetic group in cytochrome P450 and myoglobin, whereas heme c is the prosthetic group in Cytochrome C.

Heme is a coordination complex consisting of an iron ion coordinated to a protoporphyrin IX (PPIX), which acts as a tetradentate ligand, and to one or two axial ligands. Porphine, a tetrapyrrole, is a core of the protoporphyrin IX and has a marked aromatic character. Protoporphyrin IX is planar, except for the N-H bonds that are bent in the opposite direction (trans) out of the plane of the rings. PPIX contains four methyl groups, two vinyl groups, and two propionic acid groups (Figure 1). Internal and external factors modulate the redox potential of the heme cofactor of an enzyme. The redox potential of the heme group affects the chemical reaction type, and the potential of the heme group is one of the factors that determine the electron donor required to carry out the reaction. Therefore, a mechanistic understanding of the functioning of these types of enzymes in the context of the redox potential of the heme group may help to predict the type of chemical reactions these enzymes might catalyze. It can also help to engineer enzyme variants to switch the chemical reaction and enhance the enzyme efficiency for commercial applications. Physicochemical properties of the residues which are interacting with the heme cofactor, heme orientation, non-planarity of the heme, the side chain of the residue, an axial ligand which coordinates the heme iron, and the functional groups which are attached to the pyrrole rings of the heme, are the internal factors of the enzyme which affect the resting potential of the heme group in the holoenzyme. External factors such as substrates, solvent, aerobic or anaerobic environment, and electron donors also tune the redox potential of heme. The effect of functional groups attached to the porphine scaffold of the PPIX on its redox potential has been studied extensively. The orientation of one of the propionate groups towards the heme iron can stabilize the ferric heme and thus lower the redox potential. In addition, the length of the propionate has a negligible effect on the heme redox potential. In contrast, removing one propionate increases the redox potential, suggesting that the charge on the functional groups and the residues surrounding the heme may be critical for the heme redox potential [10].

## 3. Thermodynamic Modulation of Heme Redox Potential and Its Effect on the Rate of the Reaction

The Cytochrome P450 enzyme family and other heme-containing enzymes such as cytochromes, catalases, peroxidases, nitric oxide synthase, artificial metalloenzymes (ArMs), etc., catalyze a myriad of oxidative-reductive reactions, including hydroxylation, epoxidation, oxidative ring coupling, heteroatom release, heteroatom oxygenation, cyclopropanation, etc., and many of them are promiscuous in their activity [1,2,11]. Modulating the redox potential of the wild-type CYP450 enzymes by generating the variants through substitution of the residues surrounding the heme group has been explored to elucidate whether a change in redox potential could affect the chemical reaction, stereoselectivity, enzyme efficiency, compatibility with in vivo catalysis through cell transformations, switching of the electron donor, ability to catalyze a reaction in the anaerobic environment, etc. [12,13,14,15,16,17].

**Figure 2:**
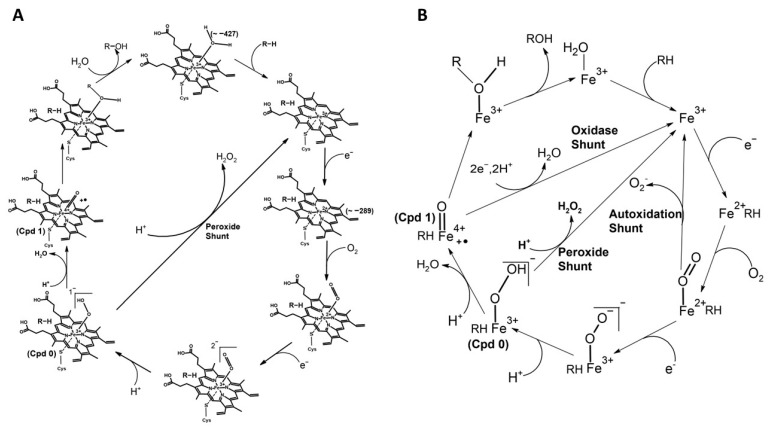
The schematic representation of the catalytic cycle of cytochrome P450. (**A**) The species (Compound 0 and Compound 1) are indicated. The species’ redox potential (mV) is given in the bracket. The axial heme ligand (cysteine thiolate, indicated as an S-atom linked to iron) and distal ligand (a water molecule that changes as the cycle progresses) are also indicated. (**B**) Uncoupling reactions with different pathways leading to the collapse of the oxy-intermediate are indicated. The oxy-ferrous form can reform the ferric state by superoxide formation via autoxidation shunt pathway. ‘Compound 0’ disintegrates with the production of peroxide and forms the ferric state (Substrate Bound) through peroxide shunt pathway. Compound 1 collapses by double reduction and diprotonation leading to the production of water via oxidase shunt pathway. These collapses can occur if there is no timely delivery of electron/proton or inappropriate positioning of the substrate.

**Figure 3:**
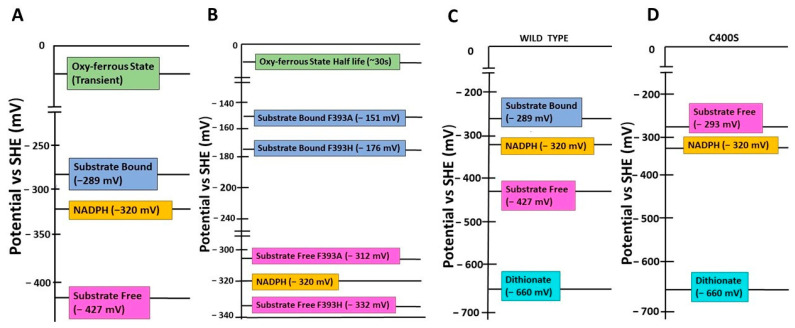
Comparison of heme redox potentials of wildtype and variants of P450_BM3_. Redox potentials (mentioned in brackets) of NADPH, substrate-free and bound heme groups, and the oxy-ferrous states of (**A**) wild-type and (**B**) the variants F393A and F393H of P450_BM3_. Redox potentials in mV (mentioned in brackets) of NADPH, dithionite, and substrate-free heme groups of (**C**) wild-type and (**D**) the variant C400S of P450_BM3_. SHE: Standard Hydrogen Electrode.

P450_BM3_, from *Bacillus megaterium*, a fatty acid monooxygenase, is well-studied and self-sufficient because the catalytic heme domain and the electron supplier diflavin reductases domain are fused in a single polypeptide. The very high catalytic activity of P450_BM3_ enzymes is manifested from the fused arrangement of redox partners and makes this system a rock star and a convenient model for mechanistic studies of P450s and P450-related enzymes on varied enzyme properties, including the redox potential of the heme group and the engineering of tailor-made variants for industrial applications [18,19]. Binding and activation of molecular oxygen are the crucial steps in the monooxygenase catalytic activity of P450 family enzymes. The reduction potential of the substrate-bound heme group regulates the rate of the reaction by modulating the above steps (Figure 2A). The reductive potential of the heme of the substrate-free wild-type P450_BM3_ enzyme (−427 mV) is much lower than the electron donor NADPH (−320 mV) (Figure 3A). Therefore, the reduction potential of the wild-type P450_BM3_ needs to be elevated to allow the electron transfer from NADPH through the reductase and ultimately to the heme. A positive gap in the reduction potential between the NADPH and substrate-bound heme group (−289 mV) of the wild-type P450_BM3_ is the driving force for the transfer of an electron from NADPH to reduce the heme group to the ferrous form and for the stabilization of this form. Molecular oxygen binds to the ferrous form of the heme group and then forms an oxy-ferrous form. Again, a positive redox potential gap between the oxy-ferrous form and the superoxy-ferric form drives the reduction of the bound dioxygen to the superoxy-ferric form (Figure 2A).

There have been studies on the roles of highly conserved F393 in P450_BM3_ on thermodynamics of enzyme activity, chemical reactions, stereo- and geometric isomerism of the product, and so on [10,20]. F393 is present within the heme-binding motif of P450_BM3_, which is positioned close to the C400, which coordinates the iron of heme and is located near to heme plane (Figure 1A). Variants of P450_BM3_ are generated by substituting F393 with an equivalent residue tyrosine and the non-equivalent residues histidine and alanine. These are used to study how the residue located at a crucial position in the heme-binding region would exert thermodynamic control over the enzyme activity by modulating the redox potential of the heme group that would affect the monooxygenase activity of the enzyme [10,20]. Catalytic competency, for monooxygenation of arachidonate and laurate substrates (measured by the turnover rate), of the F393Y variant is highly similar to the wild type, suggesting that substitution to a physicochemically equivalent residue does not affect enzyme activity. Conversely, the variants F393A and F393H are less catalytically competent than the wild-type [20]. The molar dissociation constant (KD: aka binding affinity) of the binding of these three variants to the above fatty acid substrates is not affected, suggesting that the reduction in catalytic efficiency of the variants F393A and F393H is not due to the incompetence in the substrate binding. The authors conducted further analyses to elucidate which step in the catalytic cycle could adversely affect the catalytic competency of the F393A and F393H variants. The F393A and F393H variants showed an increase in the heme iron redox potential compared to the wild-type. Conversely, the redox potential of the tyrosine-substituted variant was unchanged. Therefore, a large positive gap in the redox potential between the electron donor NADPH and the substrate-bound heme of the variants (~−151 mV for the F393A and ~−176 mV for the F393H) would increase the driving force (ΔG) for heme reduction in the variants system (Figure 3B). One could expect that an elevated redox potential of the variants could enhance the rate of heme reduction, and result in a faster turnover rate because of the faster transfer of electrons from the NADPH to the heme. Expectedly, the rate of first electron transfer to the ferric heme group of the variants F393A (1176 s^−1^ with arachidonate) and F393H (832 s^−1^) is much faster than the wild-type P450_BM3_ (348 s^−1^) [20]. Unexpectedly, the steady-state k_cat_ values of the variants (21 s^−1^ and 33 s^−1^, respectively) are much lower than the wild-type (285 s^−1^) [20].

Surprisingly, the variants exhibited a nearly 10-fold reduction in the substrate turnover, compared to the wild-type, even though the binding affinity of variants to the substrate is similar to the wild-type. The high rate of first electron transfer to the ferric heme group for its reduction has prompted authors to investigate the stability or life span of the oxy-ferrous form, where molecular oxygen is bound to the ferrous heme group and the subsequent reduction of bound oxygen leads to the superoxy-ferric form. The same magnitude (~150 mV) shift in the redox potential of both variants for substrate-bound and unbound forms suggest that the substrate has no role in modulating the reductive potential of variants compared to the wild-type [10,20]. Possibly, stage(s) after the first heme reduction would limit the turnover rate of the variants of the P450_BM3_ enzyme that do not affect the substrate’s binding. The redox potential of the substrate-bound variants is ~145 mV more than the substrate-bound wild-type, suggesting that the oxy-ferrous form is highly stable in the variants that may drastically slow down the next step of the monooxygenation reaction of P450_BM3_, that is, the reduction of dioxygen into the superoxy-ferric form. In the wild-type, the oxy-ferrous form is virtually instantaneous, and this form can be observed at cryogenic temperatures but very transiently [10]. On the contrary, the elevated reduction potential of the variants leads to the trapping of the oxy-ferrous form with a long half-life (~30 s) even at 15 °C, which decreases the rate of the overall catalysis (Figure 3B). Therefore, the architecture of the heme-binding region of P450 enzymes has evolved to have a sufficient redox potential gap between the donor and intermediate/transient species, and between the intermediate/transient and the final electron acceptor for efficient catalysis. Altering the redox potential of the heme group may elevate the rate of the formation of one of the intermediate species. However, it may decrease the formation of the following species because thermodynamic trapping of the previous intermediate leads to a reduction of the overall catalytic rate of the enzyme.

It is interesting to note that the increase in reduction potential (−427/−418 < −332 < −312 mV) of the variants correlate with the decrease (Phe/Tyr > His > Ala) in the bulkiness of the side-chain at position 393. Subsequently, Chen and co-workers have investigated the physical and structural basis of thermodynamic modulation of the heme group variants of P450_BM3_ due to a change in the bulkiness of the residue substituted at position 393 [10]. Resonance Raman spectroscopy analyses suggest that conformational changes in the vinyl groups of the heme group are the major contributor to the elevated reduction potential of the variants. A larger population of vinyl in-plane correlates with the higher reduction potential of the variants, and in turn, it correlates with the residue size at position 393. Correspondingly, smaller (Ala) residue at this position has a more vinyl in-plane population and a larger reduction potential [10]. Conversely, the bulkier (Trp) residue has a lower vinyl in-plane population and lower redox potential. Conformational changes and a network of hydrogen bond interactions of the propionate group of PPIX could also modulate the heme redox potential [10]. Substrate-binding affects the hydrogen bonding network of propionate with the residues surrounding the heme group, leading to a change in the heme redox potential. The latter is evident as an increase in the size of the residue (F393A~F393H < F393Y~WT < F393W) leads to a decrease in the heme potential, which reflects the change in the propionate conformation and its network of hydrogen bond interactions [10]. The axial ligand (residue), which coordinates the heme iron, plays a critical role in the functioning of enzymes and electron carrier proteins. Axial cysteine (C400 in P450_BM3_) thiolate ligation is essential for dioxygen activation and stabilization of the active ferryl-porphyrin cation radical oxidant during monooxygenation since it provides strong donation of electron density to the heme iron (Figure 1A and Figure 2A). Zero net change in the strength of the Fe-S bond or the γ(Fe-S) frequency in the variants suggests that despite the significant effect of the F393 substitutions on the heme redox potential, there is no effect of these substitutions on the electron density between the heme iron and the thiolate ligand [10]. Therefore, a mechanistic understanding of the structure–thermodynamic relationship of the heme by subtle variations in the residues surrounding the heme group would help to engineer the variants to modulate their functions for practical applications.

## 4. Heme Coordinated Axial Residues Altering the Heme Redox Potential and Affecting the Chemical Reaction

The very high range, −550 to +450 mV versus standard hydrogen electrode (SHE), of heme protein redox potential is one of the reasons that the heme-containing proteins participate in diverse biological functions, including a variety of chemical reactions [15]. Data accumulated over decades on these proteins revealed that heme redox potentials are modulated by a multitude of factors, including the type of porphyrin, iron (protoporphyrin IX) and its variants, the type of axial ligands, especially the residue which coordinates the heme iron, the global protein fold, polarity of the surrounding environment, etc. The advent of recombinant DNA technology and protein engineering made it easy to study the effect of the type of residues (ligands) coordinating the heme iron on the heme redox potential by using variants of the enzyme that differ from one another at the residue position which coordinates the heme iron. The effect of the substitution of heme-ligation residues on the redox potential of heme has been extensively reported using the variants [12]. In fact, the reduction potential of heme proteins can be tuned by axial ligand substitutions, and the tuning dramatically affects the thermodynamic–function relationship of the studied heme proteins [12]. The physicochemical property of the axial residue, which coordinates the heme group, plays a critical role in the redox potential of the heme. As is evident in the case of P450 enzymes, axial cysteine thiolate provides a push effect by strongly donating electron density to the heme iron [13,21,22]. Therefore, the P450 enzymes with an axial cysteine have a much more negative redox potential than heme proteins with a proximal histidine [13,14,23,24]. In myoglobin, a non-enzyme heme protein, H93 coordinates the iron of the heme in the wild-type, and the redox potential of the wild-type is ~59 mV versus the normal hydrogen electrode [NHE]. Axial ligand variants H93C and H93Y exhibited significantly lower redox potentials of −230 and −190 mV, respectively, compared to the wild-type [23].

The substitution of the proximal histidine, which ligates the heme iron by the residues cysteine or tyrosine, of different chemical properties, has converted human heme oxygenase-1 (hHO-1) to an oxidase, suggesting that tuning the redox potential of the heme through changing the iron coordinating axial residue can change the chemical reaction of an enzyme [24]. A heme-cysteine thiolate coordination active site is a common feature of many heme-containing enzymes, including P450, chloroperoxidase (CPO), and nitric oxide synthase (NOS). Heme-thiolate coordination plays a critical role in activating dioxygen or hydrogen peroxide using heme iron to insert the activated oxygen atom into various substrates. The strong electron-releasing character of the thiolate ligand has been assumed to serve as the “push” effect that decreases the redox potential of the heme and enables the O-O bond scission to generate the activated iron (IV)-oxo species commonly called Compound 1 (Figure 1A and Figure 2A) [22,25,26].

Enzymes having heme-thiolate coordination and the same porphyrin can have varying redox potentials owing to several factors, including, but not limited to, the hydrogen bond network of cysteine thiolate and the polarity of the thiolate or heme iron surrounding environment [26,27]. P450cam from *Pseudomonas putida* was used as the model to study how altering the hydrogen bond network of thiolate of C357, which coordinates the heme iron, modulates the redox potential of the heme [28]. A similar network of NH-S hydrogen bonds between the thiolate ligand and the protons from the surrounding polypeptide amides has been observed not only in other P450 enzymes such as P450eryF [29], P450_BM3_ [30], and P450terp [31], but also in non-P450 heme enzymes including CPO [32] and NOS [33]. Based on the rational substitution of residues that break the NH-S hydrogen bonds, it was found that the side-chain hydrogen bond and the electrostatic interaction of the amide proton with the thiolate ligand elevate the ~45 and ~35 mV of positive shifts, respectively, of the redox potential of the heme in P450cam [28]. The increase in the redox potential of the heme by thiolate-mediated hydrogen bonds was supported by the study by Ueno et al. which used the tetrapeptide-heme complex [34]. Altering the NH-S hydrogen bonds in the iron porphyrin-alkanethiolate complex modulates the monooxygenation activity for hydrocarbons, suggesting that these types of thiolate-mediated hydrogen bonds affect the enzyme chemical reactions mostly through changing the redox potential of the heme [35].

**Figure 4:**
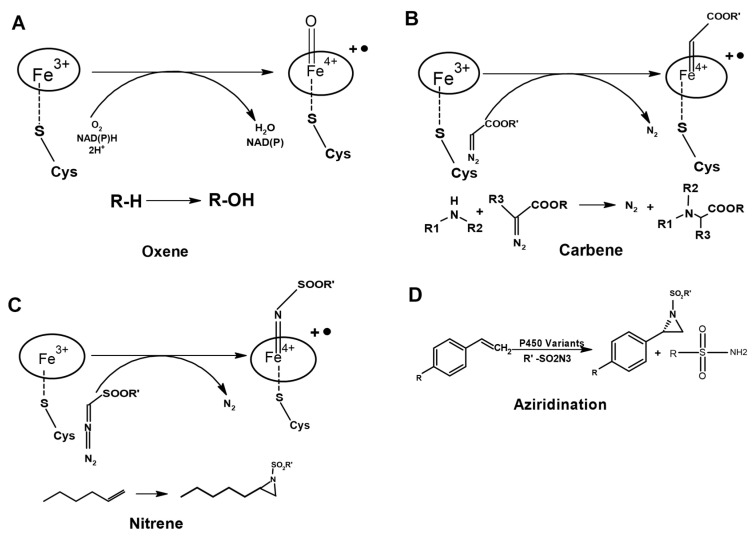
Engineering of P450s for C=C and C-H bond functionalization through abiotic transfer reactions. (**A**) The natural reaction of P450 by oxene transfer. (**B**) Carbene and (**C**) Nitrene transfer. (**D**) Aziridination with azide nitrene sources catalyzed by the variant of P450.

Oxene, carbene, and nitrene transfer using transition metal catalysts to functionalize C=C and C-H bonds have been widely used to synthesize natural product intermediates and pharmaceuticals [36,37,38], and P450 biocatalysts have been used to catalyze the above reactions [39,40,41,42]. The P450 enzymes catalyze diverse and myriad monooxygenation reactions through oxene transfer. These enzymes have been engineered to synthesize non-natural products through monooxygenation reactions [1,2,11] (Figure 4A). The wild-type P450 enzymes are promiscuous in chemical reactions since they can catalyze cyclopropanation and other reactions. They catalyze the cyclopropanation through a non-natural carbene transfer in anaerobic conditions in the presence of a non-natural reducing agent (sodium dithionite, Na_2_S_2_O_4_) (Figure 4B). However, this chemical reaction is less efficient and gives less product yield compared to a reaction with the native substrates and the native reducing agent [39]. The following changes on the P450 monooxygenase were required to make them robust cyclopropanation catalysts: (A) abolish the monooxygenease activity so that they can catalyze the cyclopropanation in aerobic conditions, (B) abolish the requirement of substrate binding to induce the transition of the P450 heme iron from low spin (E°′ FeIII/II = −430 mV) to high spin (E°′ FeIII/II = −290 mV); the high-spin heme is essential for transferring electrons from NADPH (E°′ = −320 mV) to form the oxy-ferrous form in monooxygenation reaction or ferrous-carbene form in the cyclopropanation reaction [10,17,20]. Because of a weak binding affinity (K_M_ value of ~5 mM) of non-natural substrates to the enzyme, instead of NADPH, a non-natural reducing agent such as dithionite (E°′ = −660 mV) should be used to reduce the heme. By elevation of the resting potential of the heme in the absence of the substrate would allow for the reduction of the heme by natural NADPH/NADH instead of dithionite [17] (Figure 3A,C).

The axial cysteine ligation of heme iron is essential for dioxygen activation and required for the stabilization of the active ferryl-porphyrin cation radical oxidant during monooxygenation in heme-dependent monooxygenases. On the contrary, the replacement of axial histidine in non-P450 heme oxygenase (hHO-1) by cysteine or tyrosine abolishes the monooxygenase activity of the enzyme [24]. Similarly, the substitution of axial ligated C436 to a serine residue in the case of cytochrome P4502B4 has shown the NADPH oxidase activity, but abolishes the monooxygenase activity [16]. The hemin-reconstituted C357H variant P450cam monooxygenase where cysteine is the coordinating heme iron in the wild-type exhibited NADH oxidase activity and a higher peroxidase activity by two orders of magnitude compared to the wild-type, but, there were no monooxygenase activities [14]. In addition, as discussed below, the redox potential of heme can be tuned by axial ligand substitutions, and axial cysteine to serine should raise the FeIII/II potential [17].

**Figure 5:**
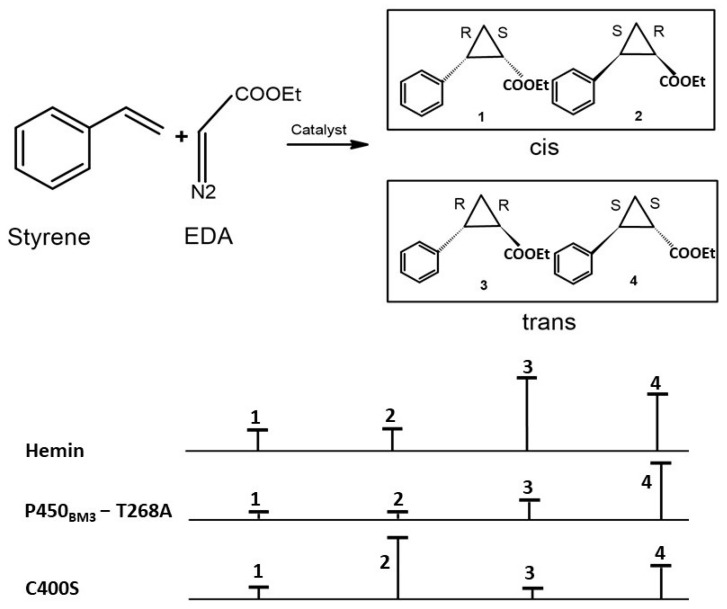
Cyclopropanation stereoselectivity of variants of P450_BM3_. The hemin and the variant T268A exhibited *trans* selectivity with a *cis*:*trans* ratio of 6:94 and 1:99, respectively. The C400S variant displays a strong preference for *cis* product formation.

Based on the above available data on modulation of the heme-containing enzyme reaction through the replacement of axial heme-coordinating residue, Coelho et al. engineered the variant P450_BM3_ to switch its activity from a fatty acid monooxygenation to robust cyclopropanation [17]. The asymmetric/enantioselective cyclopropanation of olefins with carbene precursors leads to the generation of stereoselective cyclopropanes (Figure 5), which are featured in many natural products and therapeutic agents. P450_BM3_ shows weak olefin cyclopropanation in an anaerobic condition. Thus, the authors anticipated that the substitution of C400S would maintain carbene transfer activity while eliminating monooxygenation activity, as reviewed above. The wild-type catalyzes the formation of styrene oxide through a monooxygenation reaction when NADPH is used; as expected by the authors, the C400S variant produced negligible amounts of styrene oxide, confirming the elimination of monooxygenase activity of the enzyme. On the contrary, the C400S variant was an active dithionite-driven cyclopropanation catalyst in vitro [17]. The lower binding affinity of the substrates (K_M_-styrene = 4.6 mM, K_M_-EDA = 5.7 mM) suggests that substrates of the cyclopropanation reaction are unable to raise the resting potential of the heme group upon binding to the wild-type enzyme, which is required for catalyzing the reaction. However, as anticipated, there was a rise in the redox potential of the resting state of the C400S variant by +127 mV (E°′ FeIII/II = −293 mV for the C400S variant versus −430 mV for the wild-type), which is similar to the magnitude change upon substrate binding. The latter suggests that the cyclopropanation reaction can be catalyzed by the variants with substrates of weaker binding affinity, and the heme can be reduced by NADPH (E°′ = −320 mV) instead of the dithionate (E°′ = −660 mV) to catalyze the reaction [17] (Figure 3C,D).

## 5. Elevation of Heme Redox Potential to Make In Vivo Cyclopropanation through Biotransformations

The P450_BM3_-C400S variant showed increased cyclopropanation activity compared to the wild-type when NADPH was used as the reductant under anaerobic conditions. Though P450_BM3_-C400S produced negligible amounts of styrene oxide under aerobic conditions, which confirmed the removal of monooxygenase activity, the amount of cyclopropane formed was significantly lower in aerobic conditions compared to anaerobic conditions, owing to molecular oxygen inhibition. As discussed in Section 4 of this review, the P4502B4-C436S variant exhibited two-electron oxidase activity [16], but no monooxygenase activity, suggesting that the dioxygen inhibition of cyclopropane activity of P450_BM3_-C400S could be due to the NADPH oxidase (two-electron oxidase) activity of the variant. If the P450_BM3_-C400S is to be used for the synthesis of asymmetric cyclopropanes using olefins at the commercial scale and should be economically feasible, then the reaction should be catalyzed in anaerobic conditions (Figure 5).

Synthetic wild-type and engineered enzymes have been used to genetically program the metabolic pathways in the whole cell to catalyze non-natural transformations inside cells to enable alternative routes for natural and artificial chemical product formation. Whole-cell biocatalysts have advantages over purified enzymes: they do not require further purification or isolation, have better in vivo stability, and engineered enzymes or complex assemblies which participate in the entire synthetic pathway, can be used in whole-cell biocatalysts through genetic programming. Authors have tested whether NADH can drive P450_BM3_-C400S mediated cyclopropanation as efficiently as NADPH under anaerobic conditions, so that *E. coli* whole cells expressed with P450_BM3_-C400S under anaerobic conditions, where NADPH biosynthesis does not take place, can be used as a whole-cell biocatalyst to synthesize cyclopropanes. Indeed, on a molar basis, the in vivo catalyst P450_BM3_-C400S had a turnover number (TTN) that was almost six times higher than that of the purified enzyme, under anaerobic conditions with the same stereoselectivity [17]. A catalytic TTN of more than 60,000 was observed in the in vivo reaction at high substrate loading (higher concentration of the substrates EDA and styrene) with P450_BM3_-C400S, suggesting that the whole-cell reactions are scalable to synthesize gram quantities of products. Correspondingly, whole-cell reactions were scaled to produce high quantities of cyclopropanes with high diastereo- and enantioselectivity (Figure 5). This case study is the best example of how a mechanistic understanding of the tuning of heme redox potential can be used to engineer the variants for genetic programming of whole-cell biocatalysts to produce grams of product at the scalable level for industrial applications.

## 6. Engineering P450 Variants to Switch Chemical Reactions with Enhanced Stereoselectivity and Trans-Cis Selectivity: Effect of Substitution of Residues Coordinating the Heme Group

The design and construction of enzymes as biocatalysts has been the subject of intensive studies because of their high regio- and enantioselectivities and high activities. Enzymes are highly versatile macromolecular biocatalysts that can be used to synthesize complex and pure chiral compounds with multiple stereocenters in a regio-, chemo- and stereoselective fashion. The access to non-native functions of biocatalysts can be facilitated by enzyme engineering [43]. The use of CYPs in synthesizing pharmaceuticals has also expanded due to the progress in enzyme engineering [44]. Enzyme’s catalytic pocket has evolved over millions of years to stereo- and regioselectively synthesized natural products. Redesigning them to accept even similar substrates and products with high stereoselectivity is very challenging. It is even more challenging if the recognition of one substrate allosterically affects that of the other. In addition, improving or changing more than one property (for example, high catalytic efficiency and stereoselectivity) in a single variant is a herculean task as substitutions improve one property but would negatively affect the other property. Chirality (stereoselectivity) is an important subject for academic research and pharmaceutical development. Chirality affects the physiological activity of biomolecules as (R)- and (S)-enantiomers of the racemic drug show different activities in a biological system. Indeed, researchers have been successful in the structure-based redesigning of the substrate binding pockets by selectively substituting the residue/s to cause the mutants to invert (R- to S- and vice versa) or to enhance the stereoselectivity [45,46,47,48,49]. Epoxide hydrolase, dehalogenases, etc., [50] have been successfully engineered for enantioselectivity through the iterative combinatorial active-site saturation test (CAST), directed evolution, structure-based iterative site-directed mutagenesis (ISM), and rational approaches such as Rosetta design [51]. The ‘Catalytic selectivity by computational design (CASCO)’ strategy, complemented by molecular dynamic (MD) simulation, has been used for the in silico designing of epoxide hydrolase variants library that favor binding of the substrate in a predefined form from one of the two catalytic orientations for the production of highly enantioenriched (S,S)- or (R,R)-diols [52]. The structure of R-selective alcohol dehydrogenase from *Kluyveromyces polyspora* (KpADH) has been used to identify the key residues responsible for enantioselective recognition and, subsequently, for the rational design of KpADH for enhanced R-selectivity, and also to invert stereoselectivity (to form the S-selective product) for catalyzing ketones to alcohols [53]. Using mechanism-based geometric criteria and Rosetta Enzyme Design for energy calculations, the catalytic pocket of aspartase was transformed into tailored variants of β-lyases that catalyze direct β-hydroamination of aliphatic, polar, and aromatic α,β-unsaturated carboxylic acids to form the corresponding β-amino acids with full regiospecificity and enantiospecificity [54].

The metabolism of drugs is the most stereoselective process among all the pharmacokinetic processes because drugs are metabolized by the enzymes such as P450 and uridine 5′-diphospho (UDP)-glucuronosyltransferases (UGTs) [55]. P450 enzymes are highly versatile in terms of the chirality of reactions as they exhibit substrate, product, and substrate-product stereoselectivity [55]. Structure and mechanistic understanding of P450 catalytic reactions is beneficial for predicting substrate scope and stereoselectivity of newly discovered P450s, structure-guided engineering of several biocatalysts for wider substrate specificity, and for the enhanced or inverted stereoselectivity, increased catalytic efficiency, and higher product yield.

A few case studies where the wild-type of P450s or their variants, and engineered hemeproteins are used for various chiral compound syntheses are discussed below. The Fusan and Arnold groups demonstrated that P450s and their variants could catalyze the intramolecular C-H amination of arylsulfonyl azides to form the desired sultam products with excellent regio- and stereoselectivity [56,57,58]. Non-native reactivity of a P450 enzyme P450102A1 for the cyclization of carbonazidate substrates to yield oxazolidinones via an intramolecular nitrene C-H insertion reaction has been leveraged to engineer variants of P450102A1 to amino-functionalization of two terpene natural products with high regio- and stereoselectivity to synthesize precursors of chiral amino alcohols [56] (Figure 4C). Further, mechanistic and kinetic isotope effect (KIE) experiments suggest that the C-H activation step is analogous to the native P450-mediated hydroxylation reaction, and it is the rate-limiting step that proceeds in a stepwise manner involving C-H abstraction followed by a radical rebound, which hinders the use of these enzymes for the cyclization of carbonazidate [56]. However, these mechanistic understandings, and given that these enzymes can use terpene substrates, demonstrates the feasibility of engineering the variants to improve the catalytic activity for the P450-based amination catalysis for the transformation of complex natural products such as terpenes into complex amino alcohols [56].

The greatest challenge in enzyme engineering is to create the variant to catalyze a non-native reaction with higher enantioselectivity and productivity. It is even more challenging if the substrates exhibit lower reactivity and are less water soluble. The C400S variant of P450_BM3_ (substituting the heme iron coordinating cysteine residue to serine) enhances its non-natural carbene and nitrene transfer activities [17,58,59]. Its substrate-binding pocket has also been evolved to catalyze another non-natural reaction of enantioselective intermolecular aziridination using the less soluble styrenes as substrate [58,60] (Figure 4C,D). The evolved variant exhibits high enantioselectivity (up to 99% ee) and productivity (Total Turnover number of up to 1000) for intermolecular aziridination [60], suggesting that P450 enzymes are a ‘gold mine’ to engineer/evolve biocatalysts for the catalysis of commercially viable non-natural asymmetric chemical reactions (Figure 5). The versatility in switching the chemical reaction, and the feasibility of P450 enzymes to engineer them to synthesize diverse chiral compounds, is, again, supported by the work of Prier et al., who engineered the P450_BM3_ to carry out asymmetric benzylic C-H amination on a range of substrates to synthesize valuable benzylic amines in whole cells with exceptional turnovers, exceeding the highest turnover number reported for chiral synthetic catalysts [61].

As discussed in Section 4 of this review, heme reactivity is greatly influenced by axial ligand coordination. Substitution of the residue, which coordinates iron of the heme, is detrimental to native chemical reactions but enhances the reaction rate, stereoselectivity and productivity of non-native chemical reactions, which might be through tuning of the redox potential of the heme [62]. An engineered variant of P450_BM3_ where heme iron coordinating cysteine is substituted by serine exhibited enantioselective intramolecular C-H amination in the azide substrates, affecting both the stereoselectivity and catalytic efficiency of the enzyme [58]. Similarly, the Arnold group evolved the above variant for the asymmetric synthesis of enantio-enriched and protected allylic amines using bulkier allylic sulfides, and also to synthesize the highly enantioselective imidation of non-allylic sulfides [63]. Controlling the stereoselectivity of the products in the chemical reactions is extremely challenging if the product has more than two stereocenters; therefore, products have very low enantio- and/or diastereoselectivity. Enzymes with native chemical reactions usually have higher stereoselectivity than the engineered variants for non-native reactions because they have evolved over millions of years for an asymmetric enzymatic reaction. P450_BM3_ has been engineered, through threonine to alanine substitution at position 268th, to catalyze the high diastereo- and enantioselective (cis: trans 92:8 and −97% ee-cis) cyclopropanation of styrenes from diazoester reagents via putative carbene transfer (Figure 4B), suggesting that P450 enzymes are amenable to engineering to synthesize highly stereoselective products having two chiral centers [39] (Figure 5). A new variant developed combining the C400S substitution and the substitutions from the above cyclopropanation catalyzing variant exhibited better stereoselectivity (99% ee-cis) with a similar cis-trans selectivity (Figure 5), with a Total turnover number of more than 60,000, and is readily scalable to make gram quantities of cyclopropanes in vivo whole-cell reactions. These observations suggest that the substitution of axial residue, which coordinates iron of the heme, can enhance the stereoselectivity and catalytic efficiency of the wild-type or a variant of a biocatalyst [17].

## 7. Converting Non-Enzyme to an Enzyme through Modification of the Heme Binding Pocket

Converting the non-enzyme to the enzyme to catalyze specific chemical reactions is one of the most outstanding achievements in the field of enzyme engineering. Heme is a cofactor not only in enzymes (P450, catalase, peroxidase, tryptophan oxidase, etc.) but also in many other proteins, such as in oxygen-carrying (hemoglobin and myoglobin), oxygen reduction, electron transfer (cytochromes), etc. Understanding how the same prosthetic group such as heme is used by evolution to have proteins with diverse functions would help design non-natural proteins of different functions with the same heme group. The latter would also reshape wild-type proteins to switch their function to non-native with the same prosthetic group. In this context, pioneer work from the Fusan group revealed that the oxygen-carrying heme protein myoglobin can be engineered to function as the biocatalyst to catalyze diverse chemical reactions, through substituting heme iron coordinating histidine to the equivalent residue present in the heme-containing enzymes and engineering the substrate binding pocket in myoglobin [64,65,66,67,68,69]. More remarkably, artificial metalloenzymes such as myoglobin were rationally engineered to synthesize fine chemicals and drugs at a gram scale with more than one chiral center with excellent enantiomeric (96–99.9% ee) and diastereomeric (98–99.9% de) excesses [69,70]. A cut above, the replacement of heme of the myoglobin with manganese or iron porphycene, or modification of the heme propionates, leads to myoglobin catalyzing the chemical reactions, suggesting that reconstitution of (heme) proteins with artificial porphyrins could be a strategy for non-natural functionalization such as biocatalysis [71,72,73,74,75,76,77]. P450 and myoglobin studies suggest that iron porphyrins are poor catalysts for some reactions such as C-H amination. However, slight alterations in the residues that make protein-scaffold bind the heme can confer catalytically efficient chemical reactions with higher stereoselectivity, which is impossible with wild-type proteins, enzymes, or an unreactive iron-heme factor. Since the modification of the protein scaffold, that holds the heme functional group, leads to a change in heme reduction potential that significantly affects the chemical reaction, it is imperative to study the relationship between heme redox potential and the chemical reaction in the context of engineered variants. This understanding can be used as a tool to predict the chemical reaction based on the modulation of the redox potential of heme.

Numerous protein scaffolds have been used successfully to create artificial metalloenzymes for organometallic catalysis, both for homogeneous and enzymatic catalysis. These artificial enzymes encompass a vast range of chemical reactions with excellent regio- and stereoselectivity, which highlights their importance for practical applications such as the nature-inspired design of biocatalysts and green chemistry [77,78,79]. Recently, naturally occurring metalloenzymes were repurposed to catalyze unnatural radical reactions in a stereocontrolled fashion. This represents a promising solution to tame fleeting radical intermediates for asymmetric synthesis [80].

## 8. Role of Substrates and Small Molecules in Tuning the P450-Mediated Chemical Reactions: Perspective in Commercial Applications and Enzyme Engineering

**Figure 6:**
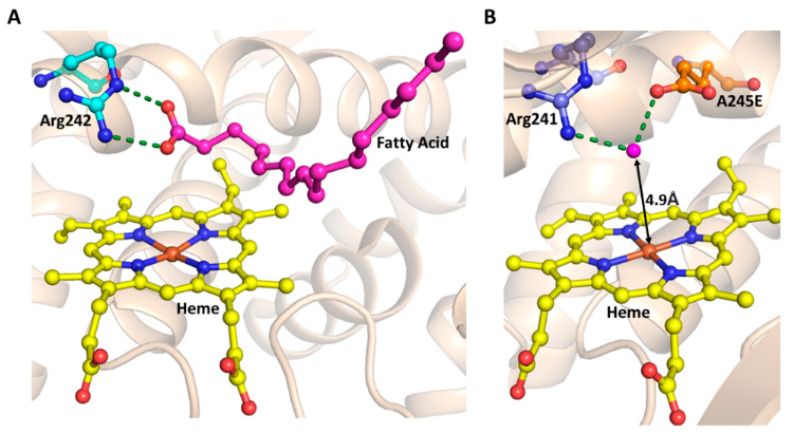
Role of the substrate and the acid-base pair in H_2_O_2_-dependent peroxidation by P450 enzymes. (**A**) Salt-bridge formation between Arg242 (cyan sticks) and Fatty Acid (magenta sticks) in P450BSβ (PDB ID: 1IZO). (**B**) Rationally engineered acid-base pair in P450SPα. The variant was engineered by A245E substitution. Interactions of E245 (orange sticks) with Arg241 (cyan sticks) are represented in broken green lines (PDB ID: 3VOO). The heme prosthetic group is shown with yellow stick representation.

In many cofactor-dependent enzymes, substrate or ligand-induced activation of enzyme activity is a common regulation mechanism used to prevent the unnecessary waste of cofactors such as ATP, NADPH, FADH, NADH, SAM, etc. [81,82,83,84]. However, mechanisms of substrates induced activation of enzyme activities depend on the enzyme system. P450s such as P450_BM3_ and P450cam catalyze the hydroxylation or decarboxylation of fatty acids through a substrate-assisted reaction mechanism, in which substrate binding elevates the redox potential of the heme group leading to normal NADPH-dependent catalysis of the reaction. Although several thousand P450 genes are reported, and biochemically characterized P450s showed wide substrates scope and variety of both natural and non-natural asymmetric chemical reactions to functionalize the C-H bonds. The need for expensive cofactors such as NADPH is one of the roadblocks to the development of these enzymes for practical biocatalyst applications [5]. The answer to this shortcoming is in the use of alternatives, such as low-cost hydrogen peroxide (H_2_O_2_) as the terminal oxidant. The peroxide shunt pathway has been reported in a few native P450s that can utilize H_2_O_2_ for the hydroxylation or decarboxylation of fatty acids through a substrate-assisted acid-base catalytic mechanism [85,86] (Figure 2B and Figure 6A). The inefficient nature of the peroxide shunt pathway compared to the NADPH-dependent pathway prevents the exploitation of this pathway for commercial applications. The X-ray crystal structure of the P450BSβ, which is functioning through the peroxide shunt pathway, complexed with a substrate palmitic acid, indicated the following unique catalytic mechanisms [86]: (A) the substrate palmitic acid acts as a co-catalyst where the terminal carboxyl group of the fatty acid forms salt bridge interactions with the arginine residue located near the heme, so that it fixes the substrates in the catalytically active position (Figure 2B and Figure 6A); (B) fatty-acid-arginine interactions are very crucial as this general acid-base functioning allows the accepting of H_2_O_2_ to initiate, as well as activating, the reaction for the facile generation of the active species Compound 1 to oxidize the substrates (Figure 2B and Figure 6A). Understanding this catalytic mechanism of the P450 enzymes lead researchers to develop strategies to adapt the P450-mediated peroxide shunt pathway for regio- and stereoselective sulfoxidation, hydroxylation, and oxidative demethylation catalytic reactions for the cost-effective use of biocatalysts, which are discussed briefly below.

**Figure 7:**
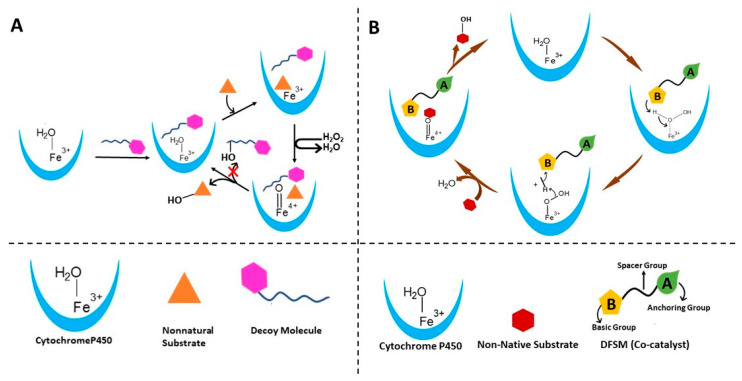
Schematics of different strategies used to tune the P450 enzymes to catalyze the H_2_O_2_-dependent oxidation reactions. (**A**) Strategy for oxidation of nonnatural substrates in the presence of decoy molecule by Cytochrome P450. This involves the addition of a dummy substrate to accommodate non-natural substrate by remolding the active site. (**B**) DFSM co-catalysis strategy that involves the addition of a co-catalyst to aid the NADPH-dependent Cytochrome P450 for activation of the peroxide pathway for the catalysis of reactions of non-native substrates.

When the alkyl chain of carboxylic acids was shorter than a length of 10 carbon atoms, no H_2_O_2_-mediated hydroxylation was observed, suggesting that these substrates could not be anchored in a hydrophobic pocket in such a way that it can make salt bridge interactions with arginine and, at the same time, the C-H group orient towards the heme group for hydroxylation [87]. Based on this, the author presumed that a shorter decoy molecule bearing the carboxyl group, which can act as a co-catalyst for acid-base catalysis, could turn over different substrates into products. In fact, peroxide-mediated enantioselective ethylbenzene hydroxylation and enantioselective styrene epoxidation of different non-natural substrates were catalyzed by the P450_BSβ_, through one-electron oxidation, in the presence of carboxyl acids whose chain length varies from C5 to C10 atoms [19,87] (Figure 7A). Catalytic rates of the above reactions varying with a chain length of the carboxylic group (maximum rate with a chain length of C5 to C7) suggested that chain length is most important for the co-catalyst/decoy molecule to participate in both the initiation and activation of the reaction [19,87]. Inspired by the decoy molecule strategies, dual-function small molecule (DFSM) co-catalysis was recently used to switch the NADPH-dependent P450_BM3_ into H_2_O_2_-driven peroxygenase activity for the development of less costly P450 biocatalyst technology (Figure 7B). As the name indicates, DFSM has two groups: an anchoring group, usually hydrophobic in nature, is connected to a basic group, which acts as a general acid-base catalyst to activate H_2_O_2_ through an appropriate length of a linker (Figure 7B). Decoy and DFSM strategies have been extensively used to alter the substrate specificity and chemical reaction of both NADPH- and H_2_O_2_-dependent P450s, suggesting that these strategies, complemented by protein engineering technology and whole-cell catalysis, would help to generate a combination of toolboxes for developing practical P450 biocatalysts for the synthesis of novel and commercial chiral chemical products [7,8,87,88,89,90,91,92,93,94,95,96,97,98,99,100,101,102,103] (Figure 7). Elsewhere, there are review articles that have extensively discussed decoy and DFSM strategies, as applied to different P450s to tune or switch the chemical reactions and to accept the non-natural substrates [6,7,96].

Another approach is to create the acid-base catalytic reaction center above the heme plane. This approach was used to (A) convert NADPH-dependent P450 to H_2_O_2_-dependent (B) H_2_O_2_-dependent oxidization of non-native substrates, independent of the decoy/DMSF molecule [8]. Ala245 of P450SPα is substituted to Glu, where the carboxylic group is located on the heme group. Here, Glu245 and Arg241 interact with a water molecule whose distance from heme is 4.9Å, which makes it suitable for the acid-base function. Here, the water molecule acts as the acid-base catalyst [8] (Figure 6B). The above variant catalyzed the styrene epoxidation independent of the decoy molecule, with a catalytic efficiency comparable to natural peroxygenases. Similarly, NADPH- dependent oxygenases P450_BM3_, P450cam, and CYP119 are turned into peroxygenases by engineering the variant of these enzymes, by replacing conserved threonine above the heme group with the glutamate [8]. The same rational enzyme engineering approach of threonine to glutamate substitution was used to engineer the variant, which exhibited a thousand-fold reduction in native monooxygenase activity but a significant improvement in H_2_O_2_-peroxygenase activity, compared to the wild-type, CYP199A4, for oxidative demethylation of 4-methoxybenzoic acid to 4-hydroxybenzoic and veratric acid to vanillic acid [104] (Figure 6B).

## 9. Conclusions

Over the decades, P450 enzyme properties have been tailored through rational, semi-rational engineering, and directed evolution approaches to catalyze the abiotic reactions, for use in genetically programmed metabolic pathways, and for chemoenzymatic processes to access novel chiral compounds that cannot be synthesized by natural or by chemical routes. Techniques such as directed evolution are advanced to tailor naturally occurring proteins for various biotechnological applications [105,106]. There is no limitation in the diversity of P450s as more than 3.5 hundred thousand sequences have been submitted in the databases. These sequences are exponentially growing, and many of them are experimentally characterized. However, wild-type P450s and newly discovered forms have obstacles for their use in synthetic biology and biotechnology applications. This is because of poor stability or solvent compatibility, need of expensive cofactors and redox partners, narrow substrate scope and chemical reaction, and poor regio- and stereoselectivity. Mechanistic understanding of the structure–function relationship of the above properties [107] would help in the rational engineering of P450s, designing artificial metalloenzymes, and converting non-enzyme heme proteins to heme enzymes to synthesize high-value chiral organic molecules. Cytochrome P450s have been engineered for a variety of purposes ranging from altered reaction types [108] to enhanced catalytic properties such as substrate specificity [109,110,111], stereoselectivity, and efficiency [80,112].

**Figure 8:**
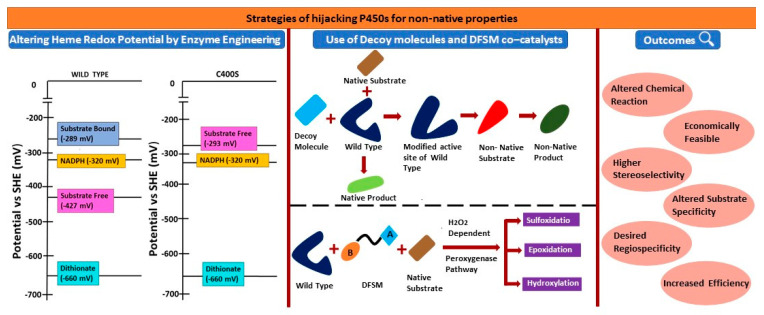
Summary of the review. Strategies for hijacking P450s for non-natural properties to alter the chemical reactions and for the asymmetric synthesis. Altering hem redox potential for non-native reactions and to switch the stereoselectivity, and employing decoy molecules and DFSM catalysts for non-native reactions and/ or for accepting non-native substrates.

In this review, we have summarized the following aspects (Figure 8): (A) how subtle variations in the residues coordinating heme and iron of the heme affect the catalytic cycle, the type of the chemical reactions, the rate of the reaction, preference of the cofactor, and stereoselectivity of the P450s; (B) how an understanding of the heme coordination by the peptide/protein frame helps to convert non-enzyme heme proteins to enzymes and to design ArMs;(C) tuning the P450-mediated chemical reactions using the substrate mimetics, decoy molecules, and dual function small molecules; (D) creating the acid-base catalytic reaction center above the heme plane to convert NADPH-dependent P450 to H_2_O_2_-dependent and H_2_O_2_-dependent oxidization of non-native substrates independent of the decoy/DMSF molecule. These aspects are essential for the rational engineering of P450s, design of ArMs, and reshaping of non-enzyme heme proteins to enzymes for synthetic and biotechnology applications [107,113,114].
